# Idiopathic Arginine Vasopressin Deficiency With Mild and Reversible Hypercalcemia

**DOI:** 10.31486/toj.24.0089

**Published:** 2025

**Authors:** Aayush Malik, Alpesh Goyal, Rahul Gupta, Abhinav Bhagat

**Affiliations:** ^1^ Department of Endocrinology and Metabolism, All India Institute of Medical Sciences, Bhopal, Madhya Pradesh, India; ^2^ Department of Radiodiagnosis, All India Institute of Medical Sciences, Bhopal, Madhya Pradesh, India

**Keywords:** *Arginine vasopressin*, *dehydration*, *diabetes insipidus*, *hypercalcemia*, *pituitary diseases*, *polydipsia*, *polyuria*

## Abstract

**Background:**

Arginine vasopressin deficiency (central diabetes insipidus) results from impaired hypothalamic-neurohypophyseal secretion of arginine vasopressin and leads to hypotonic polyuria and polydipsia. Common causes of arginine vasopressin deficiency include head trauma, pituitary surgery, neoplasms, and inflammatory stalk lesions; however, 25% to 50% of cases are idiopathic. Hypercalcemia can result in arginine vasopressin resistance (nephrogenic diabetes insipidus) and is an important differential in the evaluation of patients with hypotonic polyuria-polydipsia syndrome.

**Case Report:**

A 32-year-old male presented with polyuria (24-hour urine output of 144 mL/kg), polydipsia (24-hour fluid intake of 130 mL/kg), and nocturia of 6 months’ duration. Baseline investigations revealed normal liver, renal, serum potassium, and blood glucose levels. After overnight dehydration, serum osmolality increased to 317 mOsm/kg, while urine osmolality remained inappropriately low at 156 mOsm/kg. Mild hypercalcemia (serum calcium of 11.1 mg/dL) was noted. Upon arginine vasopressin challenge, urine osmolality increased by nearly 300%, suggesting complete arginine vasopressin deficiency. Evaluation for secondary causes was unremarkable. Magnetic resonance imaging of the pituitary revealed a normal anterior pituitary and pituitary stalk with an absent posterior pituitary bright spot. Idiopathic arginine vasopressin deficiency was diagnosed. The patient responded to oral desmopressin replacement, and normocalcemia was documented in multiple samples repeated when the patient was in a hydrated state.

**Conclusion:**

Patients with arginine vasopressin deficiency can manifest concomitant mild and reversible dehydration-related hypercalcemia. A brisk increase in urine osmolality following subcutaneous arginine vasopressin injection and normal serum calcium levels after desmopressin therapy can establish that hypercalcemia is the effect and not the cause of the primary disorder.

## INTRODUCTION

Diabetes insipidus (proposed to be renamed as arginine vasopressin deficiency or resistance) affects approximately 1 in 25,000 individuals.^1^ The disorder is characterized by hypotonic polyuria (>50 mL/kg urine output in 24 hours) and polydipsia (>3 L fluid intake in 24 hours) that impact a patient's health and quality of life.^2,3^ The major forms include (1) arginine vasopressin deficiency (also called central diabetes insipidus) caused by the impaired hypothalamic-neurohypophyseal secretion of arginine vasopressin, (2) arginine vasopressin resistance (also called nephrogenic diabetes insipidus) caused by resistance to arginine vasopressin action at the kidneys, (3) gestational arginine vasopressin deficiency caused by the accelerated metabolism of arginine vasopressin by placental vasopressinase, and (4) primary polydipsia (a thirst disorder) marked by excessive fluid intake that leads to washout of the renal medullary osmotic gradient and the development of polyuria.

Arginine vasopressin deficiency and resistance have similar clinical presentations; however, the 2 disorders differ in pathophysiology. Arginine vasopressin deficiency is caused by damage to the hypothalamus or neurohypophysis and is characterized by low circulating levels of arginine vasopressin. On the other hand, arginine vasopressin resistance is caused by medications, electrolyte imbalance, and structural kidney diseases that impair arginine vasopressin action; in patients with arginine vasopressin resistance, circulating arginine vasopressin levels are often high.^1^ Diagnosis is established through careful history and physical examination, followed by biochemical tests including a water deprivation test with arginine vasopressin challenge and hypertonic saline challenge test.^1,4^

Alternative causes of polyuria-polydipsia syndrome—such as uncontrolled diabetes mellitus, hypokalemia, and hypercalcemia—should be excluded before beginning the evaluation for arginine vasopressin deficiency or resistance. Chronic hypercalcemia can induce arginine vasopressin resistance through autophagic degradation of aquaporin-2 channels in the kidneys, resulting in polyuria and dehydration.^5,6^ On the other hand, dehydration impairs the glomerular filtration of calcium and causes hypercalcemia, thus creating a cycle of worsening dehydration, worsening renal function, and worsening hypercalcemia.^7,8^

We describe the case of a patient with idiopathic arginine vasopressin deficiency whose concomitant dehydration-related hypercalcemia made the diagnosis challenging.

## CASE REPORT

A 32-year-old male presented to the outpatient department of our tertiary care hospital with complaints of excessive thirst and frequent urination for the prior 6 months. These complaints began after a self-resolving, nonlocalizing acute febrile illness. The patient reported an intake of approximately 10 to 11 L of fluids per day, with a preference for cold fluids, and repeated nocturnal awakenings that disturbed his quality of life. At times, he tried to suppress his thirst and could limit intake to approximately 6 L per day, but limitation of liquids resulted in lethargy and symptoms of dehydration. The patient denied symptoms of dysuria and reported passing large volumes of clear urine each time. He denied a history of head trauma, pituitary surgery, headache, vomiting, visual blurring, field defects, constitutional symptoms, or symptoms suggestive of a connective tissue disorder. He had not experienced a major stressful event in the recent past, nor had he taken any medications known to cause arginine vasopressin deficiency or resistance. He had no history that suggested anterior pituitary hormone deficiency, and his family history was unremarkable.

Prior to the current presentation, the patient had been evaluated elsewhere. Diabetes mellitus was excluded with blood investigations, and empirical treatment with oral desmopressin was started. However, the patient stopped the medication after 15 days and reported to our center for a detailed evaluation.

Examination revealed stable vital parameters, height of 169 cm, weight of 81 kg, and body mass index of 28.5 kg/m^2^. Blood pressure was 130/88 mm Hg in the seated position and 122/80 mm Hg in the standing position, suggesting no noteworthy postural drop. Systemic examination, including detailed neurologic examination, was unremarkable. The patient documented a 24-hour fluid intake of 10.5 L (130 mL/kg) and a 24-hour urine output of 11.7 L (144 mL/kg).

Laboratory investigations revealed normal liver, renal, serum potassium, and blood glucose levels and normal urine microscopic examination (Table 1). High-normal serum sodium levels were suspicious for arginine vasopressin deficiency or resistance in the setting of polyuria.

**Table 1.** Laboratory Results at Baseline and After Overnight Water Deprivation

Laboratory Test	Baseline, Unstimulated State	After Overnight (8 Hours) Water Deprivation	Reference Range
Hemoglobin, g/dL	14.8	–	13.2-16.6
Hematocrit, %	43.5	–	38.3-48.6
Serum urea, mg/dL	13	15	20-40
Serum creatinine, mg/dL	0.97	1.09	0.6-1.2
Serum sodium, mmol/L	143^a^	144	135-145
Serum potassium, mmol/L	3.9	4.6	3.5-5.5
Serum uric acid, mg/dL	7.1	–	3.5-7.2
Serum total calcium, mg/dL	–	11.1	8.5-10.5
Serum phosphate, mg/dL	–	4.0	2.5-4.5
Serum aspartate transaminase, IU/L	–	38	<40
Serum alanine transaminase, IU/L		46	<40
Serum alkaline phosphatase, IU/L	–	103	30-120
Serum albumin, g/dL	–	4.4	3.5-5.2
Random plasma glucose, mg/dL	92	–	<140
HbA1c, %	5.1	–	<5.7
Serum osmolality, mOsm/kg	278^a^	317	275-295
Urine osmolality, mOsm/kg	215	156	50-1,200, depending on fluid intake
Serum free T4, ng/dL	–	1.29	0.7-1.48
Serum thyroid stimulating hormone, μIU/mL	–	0.996	0.4-4.9
Serum anti-thyroid peroxidase, IU/mL	–	1.61	<60
Serum 8 AM cortisol, fasting, μg/dL	–	9.3	3.7-19
Serum 8 AM testosterone, fasting, ng/dL	–	816.9	300-1,000

^a^Serum sodium and serum osmolality measurements at the baseline timepoint are not paired; the reported values are from 2 different days.

Baseline serum osmolality and urine osmolality were unrevealing, so stimulated measurements were planned after overnight water deprivation (dehydration) of 8 hours (2400 to 0800 hours). The dehydration measurements of serum osmolality (317 mOsm/kg) and urine osmolality (156 mOsm/kg) indicated either complete arginine vasopressin deficiency or resistance.

Evaluation for other anterior pituitary hormone deficits, including hypothyroidism, hypoadrenalism, and hypogonadism, was unrevealing. However, mild hypercalcemia was noted.

Differential diagnoses for polyuria include diabetes mellitus (osmotic diuresis), hypokalemia and hypercalcemia (secondary arginine vasopressin resistance), hyperthyroidism (increased renal plasma flow), urinary tract infections (increased frequency that may be misinterpreted as polyuria), and disorders of water balance such as primary polydipsia (washout of renal medullary gradient), arginine vasopressin deficiency (central diabetes insipidus), and arginine vasopressin resistance (nephrogenic diabetes insipidus).^1,4,9-11^

In our patient, relevant investigations excluded diabetes mellitus, hypokalemia, hyperthyroidism, and urinary tract infection. Patients with primary polydipsia have a primary thirst defect and often present with frankly low or low-normal serum sodium levels, which was not the case for our patient. On the other hand, given our patient's high-normal serum sodium level, the possibility of arginine vasopressin deficiency or resistance was strongly considered. After overnight dehydration, the patient also had mild hypercalcemia (serum total calcium of 11.1 mg/dL). However, a serum calcium measurement repeated when the patient was in a hydrated state was normal (9.96 mg/dL), with a corresponding intact parathyroid hormone and 25-hydroxyvitamin D levels of 29.7 pg/mL (reference range, 15.0-68.3 pg/mL) and 30.5 ng/mL (reference range, 20-50 ng/mL), respectively. Consequently, the possibility of hypercalcemia-induced polyuria was also considered remote.

Because of the serum and urine osmolality values after overnight dehydration, an arginine vasopressin challenge test was performed on a different day (Table 2). After obtaining baseline measurements of serum osmolality, serum sodium, urine osmolality, and urine output, 5 IU of arginine vasopressin was administered subcutaneously, and repeat laboratory measurements were obtained at 60 and 120 minutes. The patient's urine output declined from 300 mL/h at baseline to 20 mL/h at 120 minutes, and urine osmolality increased by nearly 300% at 60 minutes and by nearly 250% at 120 minutes (a >50% increase suggests complete arginine vasopressin deficiency).

**Table 2.** Laboratory Results Before and After Arginine Vasopressin Challenge Test

		After Arginine Vasopressin Administration^a^	
Laboratory Test	Before Arginine Vasopressin Administration Baseline, 0 minutes	60 minutes	120 minutes
Serum osmolality, mOsm/kg	297	–	283
Serum sodium, mmol/L	139	–	137
Urine osmolality, mOsm/kg	124	488	427
Urine output, mL/h	300		20

^a^Arginine vasopressin dose was 5 IU administered subcutaneously.

Because a diagnosis of complete arginine vasopressin deficiency was considered, magnetic resonance imaging (MRI) of the pituitary was performed. Imaging revealed a normal anterior pituitary and pituitary stalk with an absent posterior pituitary bright spot on T1-weighted images, suggesting a diagnosis of arginine vasopressin deficiency in the absence of structural pathology (Figure). Chest radiograph and blood levels of alpha-fetoprotein, beta-human chorionic gonadotropin, and angiotensin-converting enzyme were also normal.

**Figure.**
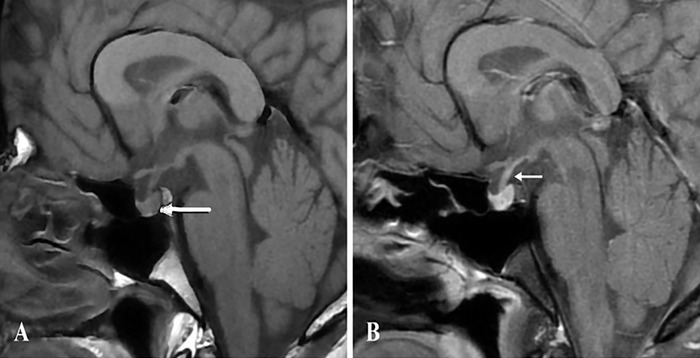
(A) Noncontrast sagittal T1-weighted image shows an absent posterior pituitary bright spot (normal location marked by white arrow). (B) Contrast-enhanced image shows normally enhancing pituitary stalk (white arrow) without any thickening.

A diagnosis of idiopathic arginine vasopressin deficiency (complete) was established, and the patient was started on an oral desmopressin (0.01 mg) tablet at bedtime with the advice to drink fluids as per thirst. The patient reported notable symptomatic improvement at a follow-up visit 4 weeks later. He was especially relieved of the nocturia and the related awakenings, thus feeling refreshed and energized in the morning. Repeat biochemistry performed at 1 and 4 months after treatment initiation revealed normal serum sodium in the 135 to 140 mmol/L range and normal serum total calcium in the 9.0 to 9.5 mg/dL range.

The patient was advised to continue desmopressin indefinitely, with a plan to repeat the pituitary MRI and the pituitary hormone profile at periodic intervals to detect any evolving structural pathology.

## DISCUSSION

In this report, we describe a case of arginine vasopressin deficiency (central diabetes insipidus) with concomitant dehydration-related mild and reversible hypercalcemia that presented a clinical challenge. Hypercalcemia is known to cause reversible arginine vasopressin resistance (nephrogenic diabetes insipidus); consequently, the possibility of polyuria being secondary to this condition was entertained. However, hypercalcemia was considered to be the effect and not the cause of polyuria because (1) serum calcium levels were mildly elevated after overnight dehydration but normalized when the patient was in a hydrated state and after the institution of desmopressin therapy, and (2) a large increase in urine osmolality was seen following arginine vasopressin injection which favors arginine vasopressin deficiency rather than resistance. Previous reports have described severe reversible hypercalcemia in settings of dehydration related to colonic ischemia and poor oral intake with concomitant gastrointestinal illness^12,13^; however, similar findings in a setting of arginine vasopressin deficiency have not been described.

Arginine vasopressin is a nonapeptide that is synthesized in the supraoptic and paraventricular nucleus of the hypothalamus and temporarily stored in the posterior pituitary.^14^ It works through a G-protein coupled receptor (V2R) present on the basolateral membrane of principal cells of renal collecting ducts. Stimulation of this receptor results in the translocation of aquaporin-2 channels from intracellular vesicles to the apical plasma membrane, thereby increasing water permeability.^15^ Deficient secretion of arginine vasopressin or resistance to its action leads to diabetes insipidus, a condition characterized by hypotonic polyuria. Patients with this condition compensate for the fluid losses through the intact thirst mechanism and therefore do not develop hypernatremia unless the thirst mechanism is compromised because of loss of consciousness or because of thirst dysregulation as in adipsic arginine vasopressin deficiency.

A working group of representatives from various international endocrinology societies has proposed changing the name diabetes insipidus to arginine vasopressin deficiency or resistance.^16^ One of the major reasons for this change is the confusion with diabetes mellitus—because of the common word *diabetes*—and resultant treatment errors. These errors include omission of desmopressin doses, frequent blood glucose tests, and initiation of antihyperglycemic treatment.^17,18^ Furthermore, the new name better reflects the disease pathophysiology, which is a welcome step in terms of scientific accuracy. To facilitate the transition and avoid confusion in scientific literature, a further recommendation of the working group is to continue to provide the prior names (central and nephrogenic diabetes insipidus) in parentheses after the new names of arginine vasopressin deficiency or resistance.^16^

The evaluation of a patient with hypotonic polyuria-polydipsia syndrome (such as diabetes insipidus) has traditionally relied on a water deprivation test with an arginine vasopressin challenge. However, the measurement of copeptin in plasma, secreted in an equimolar amount to arginine vasopressin, has emerged as a reliable tool.^19^ A baseline random plasma copeptin level ≥21.4 pmol/L can differentiate arginine vasopressin resistance from other etiologies with 100% sensitivity and 100% specificity, precluding the need for a water deprivation test.^20^ If random plasma copeptin levels are <21.4 pmol/L, copeptin should be measured after an osmotic stimulation with water deprivation and/or hypertonic saline (target plasma sodium 147 to 150 mmol/L); stimulated arginine vasopressin levels >4.9 pmol/L differentiate primary polydipsia from partial arginine vasopressin deficiency with 94% sensitivity and 94% specificity and from arginine vasopressin deficiency (partial or complete) with 94% sensitivity and 96% specificity.^20^ Because plasma copeptin measurements are not available at our center, we relied on the traditional approach that clearly indicated a diagnosis of complete arginine vasopressin deficiency. Another clue toward arginine vasopressin deficiency was the patient's high-normal serum uric acid level (7.1 mg/dL). Because of the lack of V1 receptor stimulation and consequent loss of renal uric acid clearance, serum uric acid levels are often >5 mg/dL in patients with arginine vasopressin deficiency.^21^

The etiology of adult-onset arginine vasopressin deficiency (central diabetes insipidus) includes head trauma; pituitary surgery; structural causes such as craniopharyngioma, germinoma, and metastases; and inflammatory stalk pathologies such as lymphocytic hypophysitis, Langerhans cell histiocytosis, non-Langerhans cell histiocytosis, tuberculosis, and sarcoidosis.^1^ In 25% to 50% of cases of adult-onset arginine vasopressin deficiency, a definite cause cannot be identified, and such cases (like ours) are labeled idiopathic.^1,4^ Nearly one-third of patients with idiopathic arginine vasopressin deficiency have evidence of autoimmunity in the form of autoantibodies to hypothalamic arginine vasopressin-secreting cells.^22-24^ We could not measure these autoantibodies, although anti-thyroid peroxidase antibodies tested as a surrogate marker of autoimmunity were not elevated. MRI findings include loss of the posterior pituitary bright spot, a feature common to all forms of arginine vasopressin deficiency, regardless of the etiology, with or without pituitary stalk thickening. Importantly, idiopathic arginine vasopressin deficiency may be the first sign of an evolving structural pathology,^1^ so we plan to keep the patient under close follow-up and will repeat a pituitary hormone profile and MRI of the pituitary at 6 months and then annually for the first 3 years.^1^

## CONCLUSION

As shown in this case, patients with arginine vasopressin deficiency can manifest concomitant mild and reversible dehydration-related hypercalcemia. A brisk increase in urine osmolality following subcutaneous arginine vasopressin injection and normal serum calcium levels after desmopressin therapy can establish that hypercalcemia is the effect and not the cause of the primary disorder.

## Notes

*This article meets the Accreditation Council for Graduate Medical Education and the American Board of Medical Specialties Maintenance of Certification competencies for Patient Care and Medical Knowledge*.
